# Rates of viral suppression in a cohort of people with stable HIV from two community models of ART delivery versus facility-based HIV care in Lusaka, Zambia: a cluster-randomised, non-inferiority trial nested in the HPTN 071 (PopART) trial

**DOI:** 10.1016/S2352-3018(21)00242-3

**Published:** 2021-11-26

**Authors:** Mohammed Limbada, David Macleod, Vasty Situmbeko, Ellen Muhau, Osborn Shibwela, Bwalya Chiti, Sian Floyd, Albertus J Schaap, Richard Hayes, Sarah Fidler, Helen Ayles, HPTN 071 (PopART) study team, Richard Hayes, Sarah Fidler, Nulda Beyers, Helen Ayles, Peter Bock, Wafaa El-Sadr, Myron Cohen, Virginia Bond, Susan Eshleman, Deborah Donnell, Sian Floyd, Graeme Hoddinott, Deborah Donnell, Dave Macleod, David Burns, Christopher Fraser, Lynda Emel, Heather Noble, Anne Cori, Niru Sista, Sam Griffith, Ayana Moore, Tanette Headen, Rhonda White, Eric Miller, James Hargreaves, Katharina Hauck, Ranjeeta Thomas, Mohammed Limbada, Justin Bwalya, Alwyn Mwinga, Michael Pickles, Kalpana Sabapathy, Albertus J Schaap, Mwelwa Phiri, Bwalya Chiti, Lawrence Mwenge, Rory Dunbar, Kwame Shanaube, Blia Yang, Musonda Simwinga, Peter C Smith, Nomtha Mandla, Nozizwe Makola, Anneen Van Deventer, Ephraim Sakala, Karen Jennings, Barry Kosloff, Sarah Kanema, Will Probert, Ramya Kumar, Andrew Silumesi, Tim Skalland, Krista Yuhas

**Affiliations:** a Zambart, Lusaka, Zambia; b Department of Infectious Disease Epidemiology, London School of Hygiene & Tropical Medicine, London, UK; c Department of Clinical Research, London School of Hygiene & Tropical Medicine, London, UK; d Imperial College and Imperial college National Institute for Health Research Biomedical Research Centre, London, UK

## Abstract

**Background:**

Non-facility-based antiretroviral therapy (ART) delivery for people with stable HIV might increase sustainable ART coverage in low-income and middle-income countries. Within the HPTN 071 (PopART) trial, two interventions, home-based delivery (HBD) and adherence clubs (AC), which included groups of 15–30 participants who met at a communal venue, were compared with standard of care (SoC). In this trial we looked at the effectiveness and feasibility of these alternative models of care. Specifically, this trial aimed to assess whether these models of care had similar virological suppression to that of SoC 12 months after enrolment.

**Methods:**

This was a three-arm, cluster-randomised, non-inferiority trial, done in two urban communities in Lusaka, Zambia included in the HPTN 071 trial. The two communities were split into zones, which were randomly assigned (1:1:1) to the three treatment strategies: 35 zones to the SoC group, 35 zones to the HBD group, and 34 zones to the AC group. ART and adherence support were delivered once every 3 months at home for the HBD group, in groups of 15–30 people in the AC group, or in the clinic for the SoC group. Adults with HIV who were receiving first-line ART for at least 6 months, virally suppressed using national HIV guidelines in the last 12 months, had no other health conditions requiring the clinicians attention, live in the study catchment area, and provided written informed consent, were eligible for inclusion. The primary endpoint was viral suppression at 12 months (with a 6 month final measurement window [ie, 9–15 months]), defined as less than 1000 HIV RNA copies per mL, with a non-inferiority margin of 5%.

**Findings:**

Between May 5 and Dec 19, 2017, 9900 individuals were screened for inclusion, of whom 2489 (25·1%) participants were enrolled into the trial: 781 (31%) in the SoC group, 852 (34%) in the HBD group, and 856 (34%) in the AC group. A higher proportion of participants had viral load measurements in the primary outcome window in the HBD (581 [61%]of 852 participants) and AC (485 [57%] of 856 participants) groups than in the SoC (390 [50%] of 781 patients) group (p=0·0021). Of the 1096 missing observations, 152 (13·8%) were attributable to either deaths (25 [16%] participants), relocations (37 [24%] participants), or lost to follow-up (90 [59%]); 690 (63·0%) participants had viral load results outside the window period; and 254 (23·2%) did not have a viral load result. The prevalence of viral suppression was estimated to be 98·3% (95% CI 96·6 to 99·7) in the SoC group, 98·7% (97·5 to 99·6) in the HBD group, and 99·2% (98·4 to 99·8) in the AC group. This gave an estimated risk difference of 0·3% (95% CI −1·5 to 2·4) for the HBD group compared with the SoC group and 0·9% (−0·8 to 2·8) for the AC group compared with the SoC group. There was strong evidence (p<0·0001) that both community ART models were non-inferior to the SoC group (p<0·0001).

**Interpretation:**

Community models of ART delivery were as effective as facility-based care in terms of viral suppression.

**Funding:**

National Institute of Allergy and Infectious Diseases, The International Initiative for Impact Evaluation (3ie), the Bill & Melinda Gates Foundation, National Institute on Drug Abuse, National Institute of Mental Health, and President's Emergency Plan for AIDS Relief.

## Introduction

Globally, about 38 million people have HIV, of whom 25·7 million live in sub-Saharan Africa.^1^ Despite the unprecedented scale-up of antiretroviral therapy (ART) coverage in the so-called treat all era, there are concerns over the sustainability of lifelong ART for all people with HIV due to the restricted capacity of the health-care systems.^2^, ^3^

Lifelong ART, sustained engagement in care, and adherence to ART are crucial for the UNAIDS 95-95-95 targets. Although few studies have evaluated the effect of universal treatment on long-term retention, studies published since 2019 have shown that 12 months retention following universal treatment is below that required for viral suppression, highlighting the need for targeted interventions to ensure long-term sustainability.^4^, ^5^

**Research in context**

**Evidence before this study**

Although community models of antiretroviral therapy (ART) delivery have shown promising outcomes in relation to retention in care and ART adherence, there is little of evidence on whether these models will be feasible in urban, resource-limited settings and how these non-facility based models of ART delivery perform in terms of viral suppression compared with standard of care. We searched the MEDLINE, Embase, and Global health databases from Jan 1, 2010, to Aug 21, 2019, using the search terms “ART”or “Antiretroviral therapy” AND “non-health facility based care” AND “sub-Saharan Africa”. All studies measuring at least one of the following outcomes were included: viral suppression, lost to follow-up, retention, and mortality. Several systematic reviews published have shown that community HIV programmes increase both affordability and accessibility to ART with favourable clinical outcomes in terms of optimal ART adherence, virological suppression, all-cause mortality, and loss to follow-up. However only a few of these studies have compared these models of ART delivery with conventional health-care facility or with one another, making it difficult to draw conclusions regarding the effect of the models on patient clinical outcomes. To date only a few randomised trials have reported virological suppression as an outcome measure when compared with the health-care facility for people with HIV in low-income and middle-income countries in sub-Saharan Africa. For the outcome of viral suppression, these trials have showed no evidence of a difference in viral suppression between community models of ART delivery and standard of care with an overall estimated risk difference of 1% (95% CI −1 to 4). Observational studies have also shown results broadly consistent with the randomised trials, although slightly more favourable towards community models of ART delivery, with risk differences ranging from 4% to 6%. These studies have also highlighted the need for additional studies to rigorously compare clinical outcomes between the different models of ART delivery.

**Added value to this study**

This study adds evidence to the growing literature that suggests non-inferiority of community models of ART delivery in people with stable HIV on ART in high HIV burden, low-income and middle-income countries in sub-Saharan Africa for key outcome measures of viral suppression, death, or loss to follow-up compared with current standard of care. Our findings reinforce previous assertions that decentralising ART services outside the health-care facilities into the communities using trained community health-care workers supporting drug delivery and adherence support is feasible, acceptable, and as effective as health facility-based care in ensuring viral suppression 1 year after enrolment. Our trial showed similar or better clinical and virological outcomes to other trials that compared different models of ART delivery to conventional health facility-based care. The proportion of people with HIV on ART who were virally suppressed in our three study groups was more than 95%, in line with the new UNAIDS target and compares favourably with data from multiple previous trials and cohort studies. This trial also identified challenges with regard to programmatic priorities for differentiated service delivery implementation in sub-Saharan Africa in the coming years with respect to viral load testing and monitoring and evaluation of differentiated service delivery models embedded in routine HIV service delivery.

**Implications of all the available evidence**

Our study has shown that community models of ART delivery are an effective strategy because they can complement health-care facility-based care with regards to clinical outcomes and enhance patients' ability to manage HIV. These findings support national HIV programmes scaling up differentiated service delivery models in low-income and middle-income countries in an effort to expand ART eligibility and access in the context of universal treatment. As national ART programmes strive to achieve the UNAIDS 95-95-95 targets by 2030, our findings have important clinical and public health implications for low-income and middle-income countries in that these differentiated service delivery models can overcome the challenges to ART access and retention in the midst of the weak public health infrastructure and human resource crisis. Policy makers should consider piloting, evaluating, and scaling more ambitious antiretroviral community delivery programmes that can reach higher proportions of people receiving ART.

Many national programmes are scaling up alternative service delivery approaches, known as differentiated service delivery models, to cope with the growing number of people with HIV on treatment.^6^ A range of differentiated service delivery models focusing on people with stable disease have been successfully implemented in sub-Saharan Africa allowing them to engage in care through on-going adherence support and dispensation of prepacked medications by community health workers.^7^, ^8^ They differ from conventional HIV care in the type of services provided, location and frequency of contact with the health-care system, and the type of provider involved.^2^, ^9^ These models of delivery have increasingly been recognised as safe and effective alternatives to the current standard health-care facility^10^, ^11^ and have shown promising outcomes in relation to ART adherence, viral load suppression, retention in care, loss to follow-up, and all-cause mortality, in addition to decongesting health-care facilities.^12^, ^13^ However, very few have compared differentiated service delivery models to facility health care or to one another, making it difficult to draw strong conclusions on the models' effectiveness on various patient outcomes.^2^

The HPTN 071 (PopART) trial,^14^ a community randomised trial done in 21 urban communities in Zambia and South Africa, provided evidence that a combination prevention intervention, including universal testing and treatment, can reduce HIV incidence at population level. Here, we report results from a cluster randomised, non-inferiority trial nested within the HPTN 071 (PopART) trial comparing two different community models of ART delivery with the current standard of care (SoC) in an urban setting in Zambia to gather evidence on the effectiveness of these models in relation to clinical and virological outcomes in people with HIV to guide policy makers on which models to roll out in the context of universal treatment.

## Methods

### Study design and participants

This three-arm, cluster-randomised, non-inferiority trial was nested within the HPTN 071 (PopART) trial. Details of the main HPTN 071 (PopART) trial have been described previously.^15^ Our nested study was done in a catchment population of two primary health-care facilities (with an estimated population coverage of 100 000 people per community) in Lusaka, Zambia. Each community had one public health-care facility and was divided into geographical zones (clusters) that included approximately 500 households (approximately 1400 individuals ≥16 years old). Each zone was managed by a pair of trained community HIV care providers who provided home-based HIV testing and linkage and support services. The two communities were purposely selected for this nested study because they were both randomly assigned to the PopART intervention groups, with community HIV care providers already employed to deliver HIV combination prevention package, and resembled other urban settings in Zambia and sub-Saharan Africa with respect to clinic burden HIV prevalence and population migration.

At the time of the study design (June, 2016), the two communities had an HIV prevalence of approximately 20% in adults (aged 18–44 years), with an estimated 70% of all people with HIV accessing ART. The study population included adults with HIV (≥18 years) enrolled in HIV care at the two primary health-care facilities who had stable disease. These individuals had to be on first line ART for at least 6 months with an HIV viral load of less than 1000 copies RNA/mL within the preceding 12 months, in accordance with the WHO classification. Additional eligibility criterion included living within the study catchment area and willingness to provide written informed consent. The study was approved by the University of Zambia Biomedical Research Ethics committee, Lusaka, Zambia and London School of Hygiene & Tropical Medicine ethics committee, London, UK. Permission to carry out this ancillary study was also granted by the Division of AIDS at the National Institute of Health and the Zambia National Health Research Authority, Lusaka, Zambia.

### Randomisation and masking

The unit of random assignment was a community HIV care provider zone and random allocation of zones was done before the start of this study. To achieve balance across the zones or clusters, we stratified randomisation by community and restricted the randomisation within each community on average values of key outcomes: population size, HIV prevalence, proportion of people with HIV who attend the health-care facility, and distance to the health-care facility, to ensure overall balance across the study groups. A list of 10 000 random assignments meeting the restriction criteria was created for each community, numbered 0000 to 9999. Random assignment was done publicly in both communities to select the final allocation of community HIV care provider zones to the study groups. A total of 104 community HIV care provider zones across both communities were randomly assigned (1:1:1) to one of three groups. Group 1 continue ART at the facility-based standard of care (SoC group; 35 zones); group 2 had a choice of home-based ART delivery (HBD group; 35 zones); and group 3 had a choice of being in an adherence club (AC group; 34 zones); participants in the HBD and AC groups could chose to remain in facility-based SoC. As a cluster-randomised trial of a strategy to deliver HIV care service to people with HIV within a cluster, masking of participants, community HIV care providers, and study staff was not feasible.

### Procedures

The study recruited participants from May 5 to Dec 19, 2017; follow-up continued until April 30, 2019. People with HIV attending the ART clinic were offered information about the study and their files were screened for study eligibility. Participants without a viral load result in the preceding 12 months had a blood sample collected and were asked to come back after 1 month to be rescreened for eligibility. Eligible participants were escorted to the study nurse for written informed consent. Consenting participants had their zone of residence confirmed and, based on their residential zone, were assigned to one of the study groups. They were then given the option to take up the assigned intervention or continue receiving care at the facility.^16^ Participants assigned to the SoC group continued to receive care at the health-care facility according to national guidelines (appendix p 1).

In zones randomly assigned to the HBD group, a pair of community HIV care providers visited the participants in their homes once every 3 months to provide adherence support, symptom screening using a simple checklist, and dispensed prepacked drugs. In the AC group, each zone had one club consisting of 15–30 participants who met once every 3 months at an agreed communal venue for adherence support, symptom screening, and prepacked medications delivered by a community HIV care provider pair. In both intervention groups, participants returned to the clinic at 6 and 12 months for a clinical review, ART refill, and laboratory monitoring as per national guidelines (appendix p 1). The ART supply was dispensed for 3 months at all visits and throughout the study period, all participants received the fixed-dose ART combination of tenofovir disoproxil fumarate, lamivudine or emtricitabine, and efavirenz. There were no financial incentives to participate in the study.

Clinical and follow-up visits in the intervention groups are outlined in appendix (p 1). Participants in both intervention groups were reminded of their scheduled visits by recording the dates on their care card and a text message reminder was sent to their mobile phones a week before their scheduled meeting by the community HIV care providers. In the HBD group, participants not found at home at the time of the visit were followed up by the community HIV care provider via a telephone call or text message to reschedule their home visit within a period of 5 days, provided they had adequate drug supply. In the AC group, participants who were not present during the club meeting were also followed up via a telephone call and text message and asked to come to the clinic for drug refill. During these home visits and club meetings, the community HIV care providers used study forms for standardised monitoring that included adherence counselling guidelines and a symptom screening checklist for tuberculosis and sexually transmitted infections. Participants assigned to the SoC group continued to receive standard HIV care at the facility and did not have any interaction with the study staff. Participants who missed scheduled visits or were lost to care were followed up by the clinic using routine tracing procedures (including documented follow-up home visits and telephone calls to clients and emergency contacts).

All participants had their viral loads tested at the ministry of health's designated central laboratory, Lusaka, Zambia. The study team collaborated closely with the health-care facilities to ensure that results were returned on time, and part-time volunteer workers assisted with entering viral load results into participant files. At each clinical, home, or club visit, study personnel and community HIV care providers emphasised the importance and advantages of viral load testing, described how to interpret results, and reminded participants of their upcoming visit and viral load test. Because participants were not necessarily enrolled at the time of their annual viral load measurement, some adjustment of the timing of viral load test were made to ensure that all fell within 9–15 months of enrolment. At every clinical visit, the study nurse would check when participants were due for a viral load test and order a test if needed. Participants in the intervention groups who became ill or required additional clinical services (detectable viral load >1000 copies per mL) or had symptoms suggestive of other medical conditions (eg, tuberculosis) were transitioned to clinic-based care for follow-up.

Participants in the intervention groups had their medical records kept up to date by the study staff. Data for all participants were periodically extracted from their files and the routine electronic monitoring system (SmartCare, Zambian Ministry of Health, Lusaka, Zambia) to collect clinical information, such as date of ART initiation, ART dispensing intervals, and laboratory results (viral load and CD4). Furthermore, data entered into study designed forms at each home or club visit (eg, attendance registers, drug scripts, event forms, and programme diaries) were used to measure the outcomes and processes of the study objectives, monitor the implementation of the interventions and record key contextual factors. At the end of the study, all the participants in the intervention groups were transitioned to SoC.

### Outcomes

The primary endpoint was the proportion of participants with virological suppression at 12 months (with a 3 month reporting cutoff window [ie, 9–15 months]) after study enrolment. Virological suppression was defined as no more than 1000 HIV RNA copies per mL; viral load measurements were done at a designated Ministry of Health laboratory with COBAS TaqMan HIV-1 version 2·0 assay (Roche, Basel, Switzerland) at 6 months and 12 months after ART initiation and annually thereafter. If no primary outcome measurement was taken within the 3 month window then the primary outcome was considered to be missing.

The prespecified secondary endpoints were, first, the proportion of participants who were virally suppressed at 20–24 months after enrolment (as measured by last viral load result taken between 20 and 24 months after enrolment). Second, the proportion of participants lost to follow-up 12 months after enrolment, defined as having no contact more than 90 days after last missed scheduled appointment with unknown outcomes or the proportion of participants who were no longer retained on treatment with unknown outcomes after study enrolment. Participants who transferred out of the health-care facility were not considered lost to follow-up but terminated from the study; other reasons for study termination included death, lost to follow-up, and study withdrawal. Third, the proportion of participants who had died 12 months after enrolment due to any cause. Lastly, the proportion of participants retained on treatment 12 months after enrolment (defined as a documented drug pick up between 9 and 12 months after enrolment). Participants who moved to another zone with a different intervention or outside the study catchment area but continued to receive care at the health-care facility were considered retained in care. Additionally, the study recorded retention in the allocated model of care defined as the proportion of people retained in their originally allocated group. For this outcome, participants were considered non-retained in the models of care if they transitioned back to SoC for any reason, including comorbidities, lost to follow-up, death, opting out of the intervention, or withdrawal. In both intervention groups, death was recorded as reported by the community HIV care providers who delivered the interventions, whereas in the SoC group deaths were recorded from either SmartCare database or participant clinic records. All primary and secondary outcomes were assessed according to the intention-to-treat principle. No clinical adverse events were anticipated; social harms were reported using social harm forms.

### Statistical analysis

On the basis of the data derived from the HPTN 071 (PopART) trial, the number of adults with HIV who were receiving ART averaged approximately 50 per zone, with a harmonic mean of approximately 36 per zone.^16^ Assuming that 80% of the eligible adults who agreed to participate in the study, were not lost to follow-up, and had a primary endpoint measurement the number of study participants per zone would be 30. Our study power calculations using this assumption, given 104 zones randomly assigned to the three groups, gave an estimated overall sample size of 3120 participants, approximately 1040 per group. We assumed that of the study participants in the SoC group the proportion who were not virally suppressed 12 months after enrolment to the study would be between 10 and 15%.^16^ We defined the non-inferiority margin to be 5%, based on clinical judgement as to what would be a meaningful increase in non-suppression and by similar trials.^17^, ^18^, ^19^ Assuming the coefficient of variation (k) to be 0·3, the estimated study power was 91% to show that the HBD and AC groups were not inferior to the SoC group. The value of k was based on an estimate range of cluster prevalence from 4% to 16%, and the corresponding intracluster coefficient was 0·01. The power calculations used the formula for cluster-randomised, non-inferiority trials by Hayes and Moulton.^20^

Data analysis was done following the methods outlined by Hayes and Moulton.^20^ For our primary analysis, the prevalence of viral suppression in each zone within each group was estimated and the mean of the zone-specific values was calculated for each group, along with its corresponding 95% CI. Given the high prevalence of viral suppression in the primary outcome, the CI for the prevalence estimates were obtained with bootstrap methods (ie, taking 100 000 samples of size N from the zone means, calculating the mean from each of these samples, and taking the 2·5 and 97·5 percentiles). The difference in the prevalence between the groups provided the risk difference. The evidence for a difference was assessed using a one-sample t-test, with a non-inferiority margin of 5%. Because of the high prevalence of the primary outcome, bootstrap SEs were used for estimating the 95% CI of the risk difference. The proportion of bootstrap samples that showed a risk difference of more than 5% (favouring SoC groups) provided the p value for testing this hypothesis of non-inferiority. Cluster-level analysis was done to provide estimates of prevalence differences. Because results were frequently absent or delayed, with a turnaround time of 4–12 weeks, a high proportion of individuals did not receive a viral load measurement within the prespecified window. Therefore, a post-hoc sensitivity analyses of the primary outcome were done, widening the window initially to 9–18 months and then to 9–24 months. Due to the large amount of missing viral load data, an additional sensitivity analysis was done (not pre-specified in the protocol) to provide the worst case scenario in which those without a viral load result were categorised as unsuppressed. The trial was registered with ClinicalTrials.gov, NCT03025165.

### Role of the funding source

The funders had no role in the study design, data collection, data analysis, and interpretation, or writing of the report.

## Results

**Figure 1:**
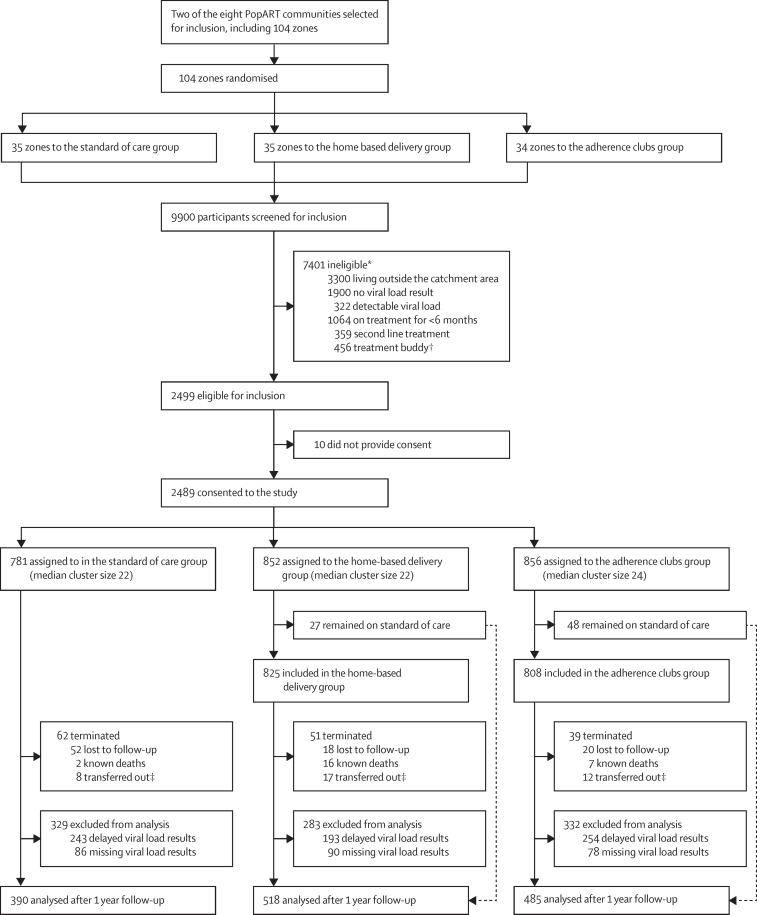
Trial profile Community one had 54 zones and community two had 50 Zones. *Based on crude estimates. †Treatment buddies are also known as treatment supporters; they support treatment (eg, by picking up drug refills if an individual with HIV cannot come to the clinic; treatment buddies were not included in our investigation. ‡Patients transferred out of the community and sought care in another health care facility.

Between May 5 and Dec 19, 2017, a total of 9900 participants were screened for eligibility in the health-care facilities across both communities. 2499 (25·2%) people with stable HIV were identified as eligible for inclusion, of whom 2489 (99·6%) consented to participate (figure 1). 1757 (70·6%) participants were female, which reflects the population of individuals on ART with stable HIV (table 1). 781 (31·4%) participants were assigned to the SoC group, 852 (34·2%) to the HBD group, and 856 (34·4%) to the AC group. 27 (3%) of 852 participants in the HBD group and 48 (6%) of 856 participants in the AC group chose to continue receiving care at the clinic. The median age of participants was 40 years (IQR 33–47) and the median duration on ART was 4 years (IQR 2–7).

**Table 1:** Baseline clinical characteristics of participants in the intervention and control arms

	**Standard of care group (n=781)**	**Home-based delivery group (n=852)**	**Adherence clubs group (n=856)**
**Communities**			
Community 1	365 (47%)	418 (50%)	370 (43%)
Community 2	416 (53%)	434 (51%)	486 (57%)
**Sex**			
Male	226 (29%)	247 (29%)	259 (30%)
Female	555 (71%)	605 (71%)	597 (70%)
**Age groups (years)**			
18–24	32 (4%)	43 (5%)	36 (4%)
25–34	190 (24%)	216 (25%)	204 (24%)
35–44	312 (40%)	342 (40%)	338 (39%)
45–54	175 (22%)	190 (22%)	189 (22%)
≥55	72 (9%)	61 (7%)	89 (10%)
Median age (years)	40 (34–47)	39 (33–46)	40 (34–47)
**Years on ART**			
<1 year	23 (3%)	30 (4%)	24 (3%)
1–2 years	214 (27%)	223 (26%)	233 (27%)
3–5 years	262 (34%)	285 (34%)	281 (33%)
≥6 years	282 (36%)	314 (37%)	318 (37%)
Median years on ART	4 (2–7)	4 (2–7)	4 (2–7)

Data are n (%) or median (IQR). Data are for the modified intention-to-treat analysis. ART=antiretroviral therapy.

**Figure 2:**
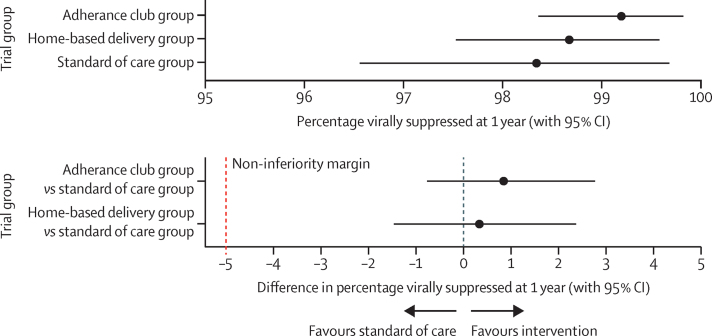
Comparison of standard of care with home-based delivery and adherence clubs (A) Estimated viral suppression in the three treatment groups. (B) Risk difference of viral suppression between the standard of care group and the two intervention groups.

1393 (56·0%) of the 2489 participants included across all three groups had a viral load result available for analysis within the 9–15 month window used for the primary outcome of viral suppression at 12 months. A higher proportion of participants had a viral load measurement in the HBD (518 [61%] of 852 participants) and AC (485 [57%] of 856 participants) groups than in the SoC group (390 [50%] of 781 participants; table 2). Of those with a viral load measurement available in the primary endpoint window, across all three groups 16 (1·1%) of 1393 were not virally suppressed. The median viral load for those who were unsuppressed was 12 870 RNA copies per mL (IQR 2175 to 28 221). Viral load suppression was estimated to be 98·3% (95% CI 96·6 to 99·7) in the SoC group compared with 98·7% (97·5 to 99·6) in the HBD group and 99·2% (98·4 to 99·8) in the AC group. The intracluster correlation coefficient was 0·01 (95% 0·00 to 0·04). This resulted in an estimated risk of viral load suppression being 0·3% (95% CI −1·5 to 2·4) for the HBD group compared with the SoC group and 0·9% (−0·8 to 2·8) for the AC group compared with the SoC group (figure 2). The lower bound of the two-sided 95% CI for both the risk differences were more than the non-inferiority margin of −5%. There was strong evidence (p<0·0001) that both the HBD and AC interventions were non-inferior to SoC, using our predefined non-inferiority margin of 5%.

**Table 2:** Viral suppression at different time points

	**Participants with viral load result**	**Participants with viral load >1000 copies per mL (IQR 2175–18 221)**	Estimated prevalence of viral suppression*(%; 95% CI)	**Risk difference *vs* standard of care**†
**9–15 months (primary outcome)**				
Standard of care group	390/781 (50%)	6/390 (2%)	98·3% (96·6 to 99·7)	..
Home-based delivery group	518/852 (61%)	6/518 (1%)	98·7% (97·5 to 99·6)	0·3% (−1·5 to 2·4)
Adherence clubs group	485/856 (57%)	4/485 (1%)	99·2% (98·4 to 99·8)	0·9% (−0·8 to 2·8)
**9–18 months**				
Standard of care group	526/781 (67%)	8/526 (2%)	98·0% (96·3 to 99·5)	..
Home-based delivery group	621/852 (73%)	10/621 (2%)	98·3% (97·3 to 99·3)	0·3% (−1·5 to 2·3)
Adherence clubs group	576/856 (67%)	7/576 (1%)	98·8% (97·9 to 99·6)	0·8% (−0·9 to 2·7)
**9–24 months**				
Standard of care group	633/781 (81%)	8/633 (1%)	98·4% (97·0 to 99·6)	..
Home-based delivery group	711/852 (83%)	13/711 (2%)	98·2% (97·2 to 98·2)	−0·2% (−1·7 to 1·5)
Adherence clubs group	739/856 (86%)	10/739 (1%)	98·6% (97·7 to 99·3)	0·2% (−1·3 to 1·8)
**20–24 months**				
Standard of care group	123/781 (16%)	2/123 (2%)	99·2% (98·0 to 100)	..
Home-based delivery group	197/852 (23%)	3/197 (2%)	98·9% (97·7 to 100)	−0·3% (−1·9 to 1·3)
Adherence clubs group	379/856 (44%)	6/379 (2%)	98·7% (97·7 to 99·6)	−0·5% (−1·9 to 1·0)

Data are n/N (%).

* Estimated prevalence based on mean of zone (cluster) prevalence's; virological suppression was defined according to the Zambian standard of care guidelines: less than 1000 HIV RNA copies per mL (based on the parameters of any assay performed through routine laboratory monitoring).

† Is the difference in the risk of virological failure between the intervention and standard of care.

Of the 1096 (44%) participants without a viral load in the primary endpoint window, 25 (2·3%) had died, 37 (3·4%) transferred out of the community, and 90 (8·2%) were lost to follow-up. Of the remaining 944 participants who did not have a viral load data recorded in the primary endpoint window, had not died, had not been transferred out of the community, and were not lost-to-follow-up, 690 (73%) had a viral load measurement taken between 15 months and 24 months (giving a total of 2083 [83·7%] participants who had a viral load result between 9 and 24 months) and 254 (10·2%) never had a viral load result, but were not lost to follow-up, had not transferred, and were not known to have died. Reasons for not having a viral load result included not having had a viral load test done, missing results, delayed processing of viral load samples, and delayed entry of viral load results into participant files and Smartcare database.

Post-hoc sensitivity analyses were done to allow the inclusion of some participants with viral load data available outside of the predefined primary endpoint window (9–15 months). First, the window was widened to allow an observation of viral load between 9 and 18 months, resulting in 1723 (69·2%) participants being included in the analysis. A second expansion of the window to 24 months resulted in the inclusion of 2083 (83·7%) participants. Across all scenarios, the proportion of participants who were virally suppressed remained very high (>98% in all groups) with strong evidence (p<0·0001) of non-inferiority (appendix p 2).

The proportion of participants retained and known to be virally suppressed at 12 months was compared in all participants across all three groups, excluding the 37 participants who were known to have transferred out of the community. The mean cluster prevalence was 50·3% (SD 14·2%) in the SoC group, 57·1% (SD 17·7%) in the AC group, and 62·3% (13·9%) in the HBD group (appendix p 3). The HBD intervention resulted in higher known viral suppression than SoC, with an estimated risk difference of 12·0% (95% CI 5·3 to 18·7; p=0·00066); although the AC intervention was also better than SoC the difference was not statistically different (risk difference 6·7% [95% CI −0·9 to 14·4]; p=0·085). In both the HBD and AC groups there was very strong evidence (p<0·0001) of non-inferiority against the 5% non-inferiority margin.

For our secondary endpoint of viral suppression in those who had a viral load result 20–24 months after enrolment, more viral load results were obtained in the AC group (44·3%) than in the HBD (23·1%) or SoC arms (15·7%). Viral suppression was estimated to be 99·2% (95% CI 98·0–100·0) in the SoC group compared with 98·9% (97·7–100·0) in the HBD group and 98·7% (97·7–99·6) in the AC group (table 2). This resulted in the estimated risk of viral suppression being slightly lower in both the HBD group and the AC group compared with the SoC group, but still well above the non-inferiority threshold of −5% (p<0·0001).

Deaths at 12 months are reported in table 3. In the entire follow-up period of 24 months, 33 (1·3%) of 2489 participants were known to have died: two (<1%) of 781 in the SoC group, 19 (2%) of 852 in the HBD group, and 12 (1%) of 856 in the AC group (table 3). Information obtained on the cause of death was mostly non-specific. In the HBD group, of the 19 participants who died, three died due to HIV-related causes, seven due to non-HIV related causes, and nine due to unknown cause. In the AC group, one died due to HIV related cause, four due to non-HIV related causes, and seven due to unknown causes. Of the two participants who died in the SoC group, neither were known to be due to HIV-related causes.

**Table 3:** Lost to follow-up and mortality across the study groups

	**Standard of care group (n=781)**	**Home-based delivery group (n=852)**	**Adherence clubs group (n=856)**
**Loss to follow-up**			
Loss to follow-up at 12 months	72 (9%)	28 (3%)	28 (3%)
Risk difference *vs* standard of care group	..	−6·4% (−9·3 to −3·5)	−6·7% (−9·7 to −3·8)
Loss to follow-up at 24 months	127 (16%)	51 (6%)	46 (5%)
Risk difference *vs* standard of care group	..	−10·9% (−14·3 to −7·6)	−11·8% (−15·3 to −8·3)
**Mortality**			
Known died at 12 months	2 (<1%)	18 (2%)	8 (1%)
Known died at 24 months	2 (<1%)	19 (2%)	12 (1%)
Combined death and lost to follow-up	129 (17%)	70 (8%)	58 (7%)

Data are n (%) or % (95% CI).

*Information obtained on the cause of death was mostly non-specific.

By the end of the study, 224 participants were lost to follow-up: 127 (57%) from the SoC group 51 (23%) from the HBD group, and 46 (21%) from the AC group. 2167 (87·1%) of 2489 participants who were retained in care at 12 months. Retention in care was highest in the AC group (776 [91%]), followed by the HBD group (745 [87%]), and last the SoC group (776 [83%]). 92 (11%) of 825 participants in the HBD group and 54 (7%) of 808 participants in the AC group were transitioned to SoC within the first year (table 4). 733 (89%) of 825 participants were retained in the HBD group at 12 months and 754 (93%) of 808 participants were retained in the AC group (table 4). Five (0·5%) participants developed tuberculosis (four in the AC group and one in the HBD group). Throughout the study period, there were no reports of adverse events or social harms.

**Table 4:** Retention in allocated model of antiretroviral delivery

		**Standard of care group (n=781)**	**Home-based delivery group (n=852)**	**Adherence clubs group (n=856)**
Chose the model assigned		781 (100%)	825 (97%)	808 (94%)
Retained in care at 12 months*		646 (83%)	745 (87%)	776 (91%)
Retained in the model of care at 12 months†		..	733 (88%)	754 (93%)
Transitioned back to standard of care within the first year after enrolment		..	92 (11%)	54 (7%)
Reasons for transition				
	Moved out or relocated out of the zone or community‡	..	53/92 (58%)	26/54 (48%)
	Opted out of the model	..	24/92 (26%)	19/54 (35%)
	Staff decision	..	15/92 (16%)	9/54 (16%)

Data are n (%) or n/N (%).

* Defined as participants who had a drug refill within the 120 days in the run up to 12 months after enrolment (ie, between 245 and 365 days after enrolment).

† Participants who were still receiving care via the intervention models and had not transitioned to standard of care.

‡ Moved out of the zone into another zone offering a different intervention or out of the community but still receiving care at the same health care facility.

## Discussion

This cluster-randomised, non-inferiority trial done in a high HIV prevalence setting in Zambia provides evidence that two community models of ART delivery were non-inferior to the current standard of care in terms of viral suppression 1 year and 2 years after enrolment. The proportion of participants with viral suppression in our three study groups was more than 95%, which compares favourably with results from other published studies and is higher than we had anticipated, partly due to the eligibility requirement of being virally suppressed within the 12 months before trial enrolment and because the median time on ART in all three arms was 4 years. Although only 55% of individuals had a viral load result during the predefined window period, sensitivity analysis including 85% of the data gave the same result.

Our study adds to the growing body of literature that streamlined services for people with stable HIV, delivered by trained community health workers to support adherence and drug delivery, is as effective as care in health-care facilities in ensuring ART adherence and viral suppression. Randomised studies from Tanzania, Uganda, and Kenya have all shown that home-based ART delivery can achieve similar or higher viral suppression and retention rates than conventional facility-based care.^18^, ^19^, ^21^ Regarding adherence clubs, our findings support randomised studies from South Africa, in which 12 month viral suppression rates were similar between adherence clubs and the health-care facilities.^22^, ^23^ However, in most of these published studies, it was difficult to ascertain the viral load coverage because the authors did not specifically describe what percentage of participants did not have a viral load available for analysis. Studies from Lesotho and Zimbabwe on multi-month dispensing and community adherence groups found no difference in viral suppression rates between community models and facility care, despite the limitations of the study from Zimbabwe in viral load results availability, which were similar to ours.^12^, ^24^ The findings of our study were also consistent with our systematic review, published in 2021, which found no evidence of differences in viral suppression between patients assigned to various forms of differentiated service delivery models and the health-care facility.^25^

In line with previous studies, retention in both intervention groups was high despite the high patient mobility in urban settings.^14^ We found no evidence of a difference in all-cause mortality rates between those assigned to the HBD group and those assigned to the AC group, although comparison of mortality rates to the SoC group was probably subject to ascertainment bias because we relied on routine clinical data, in which deaths outside the clinic are poorly recorded and information obtained on the cause of death mostly non-specific.^10^ Lost to follow-up rates in the health-care facility were significantly higher compared with both the HBD and AC groups, but this difference could have been an overestimation because death was poorly recorded, or participants could have transferred without the knowledge of the health-care facility (also known as silent transfer), with others discontinuing therapy.^18^, ^21^, ^23^, ^26^ Like many programmes in sub-Saharan Africa reporting lost to follow-up, ascertaining the actual outcomes of people who were lost to follow-up who could frequently not be traced was difficult.^27^

Our findings highlight the suboptimal routine viral load monitoring for people with HIV in low-income and middle-income countries. In our trial and a randomised study in Lesotho,^12^ non-availability of viral load results due to prolonged testing turnaround time and missing results resulted in a significant proportion of participants being ineligible for differentiated service delivery inclusion. Nearly half of our study participants were excluded from the primary analysis, and, in comparison with the standard of care, viral load coverage in both community ART models was around 5–10% better because of the viral load demand created by the study team. Although viral load testing capacity has increased in low-income and middle-income countries, insufficient testing, inadequate personnel, inefficient cold chain transportation, and weak sample referral mechanisms continue to prevent people with HIV from getting these tests.^9^, ^28^ People with an unsuppressed viral load need to be followed up by peer educators, but this approach has not been robust, and patients are more likely to be informed of their results at the next clinic appointment. There is need to strengthen laboratory services by creating an efficient feedback system, developing guidelines, and providing ongoing training and support to health-care workers about the importance of viral load monitoring and considering alternative viral load technologies, such as point-of-care viral load tests. Additionally, demand for viral load testing must be created by empowering patients to understand the significance of the test, participate in their treatment decisions, and benefit from the use of their results.^29^

During the study, ART stock-outs occurred due to health system issues or supply chain flaws, resulting in 1–2 months ART refills instead of 3 months. This might have effected adherence in the SoC group because more frequent pharmacy visits were required, underlining the need for alternative drug delivery mechanisms in low-income and middle-income countries.

Our study had several strengths in that it used a robust, cluster-randomised design to explore participant outcomes of different ART delivery models and compare them with the health-care facilities in a real-world urban setting, providing evidence that could be generalised to other in low-income and middle-income countries. The use of routine clinical and laboratory data helped prevent this study from influencing participant clinical outcomes. This study showed the acceptability of community health workers to deliver ART, despite potential stigma concerns, this might be because the repeated home visits over 3 years during the main HPTN 071 (PopART) trial solidified their relationship with the communities which could, in turn, have helped overcome many of the challenges people with HIV face in accessing care.^30^, ^31^, ^32^, ^33^

The study had several limitations. First, there could have been ascertainment bias for some outcomes, because we knew what occurred to participants in the intervention groups but not in the SoC group, restricting our ability to draw specific comparisons regarding deaths and opportunistic infections. Second, delayed viral load data excluded many potential eligible individuals from the study. The number of participants with a viral load result in the primary endpoint window was substantially lower than predicted and was lower in the SoC group than in the HBD and AC groups. This difference could have introduced bias into the results if the reason for a missing viral load was associated with viral suppression. However, the sensitivity analysis that allowed us to include many of the delayed viral load results gave us a similar result to the primary outcome. Third, we had lower recruitment in the SoC group. This imbalance might have occurred due to participant awareness of the interventions in their residential zones, leading them to visit the clinic outside their scheduled appointments to be screened for study inclusion. This might have led to overestimating viral suppression in the intervention groups. Fourth, compared with the SoC group, lost to follow-up rates were lower in the intervention groups. However, it is unlikely that those who were lost to follow-up had a higher prevalence of virological suppression than those who were not lost to follow-up, implying that increased lost to follow-up rates in the SoC group makes our comparison more conservative. Fifth, our assumption on prevalence on non-suppression was too high and despite not recruiting our target sample size, the study power was retained by the lower level of non-suppression. Finally, because participants had to be clinically stable, they were probably highly adherent. This calls into doubt their representativeness of all people with HIV and the generalisability of the results. Whereas, people who struggle with adherence and appointment keeping might benefit the most from flexible models of care and should be included in such trials.

Our study has shown that differentiated service delivery models are feasible, acceptable, and do not compromise clinical outcomes for people with stable HIV. They can overcome barriers to ART access despite the weak public health infrastructure, restricted human resources, and the day-to-day realities of living with HIV. They might even be more suited for people with less stable HIV who face difficulties attending the clinic for work, family, or stigma reasons, and offer more individualised care and peer support for newly diagnosed individuals. To fully assess the effectiveness of the models in practice, more research or programmatic evaluations are required to understand their implications long-term (5–10 years follow-up), and which factors have the greatest influence or effect on the models' effectiveness need to be determined. Exploration of annual clinic visits for people with stable HIV, in which viral load testing can be done outside the clinic, might have a beneficial effect on the cost, cost-effectiveness, and acceptability of HIV interventions on a larger scale.

In conclusion, community models of ART delivery were as effective as facility-based care in terms of viral suppression in this urban setting in Zambia. However, in settings with poor viral load resources, such frequent viral load monitoring in people receiving ART with stable HIV might not be optimal compared with efforts to enhance retention on ART or viral load monitoring in populations at higher risk of non-suppression.

## Data sharing

Research data that underpins analysis outlined in this paper cannot be made available. The dataset contains measurements that can be used to reidentify study participants, due to the number and type of variables captured. Participant and ethical consent for wider sharing was also not obtained, due to the research being done before data sharing became the norm. However, the study team invite interested parties to contact the corresponding author to discuss the research and data collected in more detail.

## Declaration of interests

HA reports grants from The Bill & Melinda Gates Foundation, National Institutes of Allergy and Infectious Diseases (NIAID), National Institute of Mental Health (NIMH), National Institute on Drug Abuse (NIDA), International Initiative for Impact Evaluation (3ie), and the US President's Emergency Plan for AIDS Relief (PEPFAR) during the study; is a member of the technical review panel for the Global Fund to Fight AIDS, Tuberculosis, and Malaria; and reports honoraria payment from global fund outside the submitted work. SFi reports grants from The Bill & Melinda Gates Foundation, NIAID, NIMH, NIDA, and 3ie during the study; is affiliated with the clinical trial HIVCORE006, St Marys Development Trust board, SHM Foundation charitable trust board; and SFi reports consulting fees from Immunocore, outside the submitted work. SFl and DM reports grants from The Bill & Melinda Gates Foundation, NIAID, NIMH, NIDA, and 3ie during the study. All other authors declare no competing interests.
