# Supplementary material for: Rates of viral suppression in a cohort of people with stable HIV from two community models of ART delivery versus facility-based HIV care in Lusaka, Zambia: a cluster-randomised, non-inferiority trial nested in the HPTN 071 (PopART) trial
Source: Lancet HIV. 2021 Nov 26;9(1):e13–23. doi: 10.1016/S2352-3018(21)00242-3 (PMC8716341; doi:10.1016/S2352-3018(21)00242-3)
Supplement: Supplementary appendix [file mmc1.pdf]

THE LANCET
HIV

Supplementary appendix

This appendix formed part of the original submission and has been peer reviewed. We post it as supplied by the authors.

Supplement to: Limbada M, Macleod D, Situmbeko V, et al. Rates of viral suppression in a cohort of people with stable HIV from two community models of ART delivery versus facility-based HIV care in Lusaka, Zambia: a cluster-randomised, non-inferiority trial nested in the HPTN 071 (PopART) trial. *Lancet HIV* 2021; published online Nov 26. http://dx.doi.org/10.1016/S2352-3018(21)00242-3.

Appendices

Appendix 1: Broad overview of the three ART delivery models.

	When	What	By Whom	Where
Model				
Standard of Care (SoC)	Month 0 (enrolment)	Clinical consultation, screening for eligibility*, consent to participate in study*<br>Drug: ART x 90 days	Clinician**	ART clinic
	Months 3, 6, 9 and 12	**Clinical visit:** Clinical consultation, adherence support[!]**,** laboratory monitoring (VL testing) [ⱡ]<br>**Drugs**: ART refill for 90 days***		
Home-Based Delivery (HBD)	Month 0 (enrolment)	Clinical consultation, screening for eligibility*, consent to participate in study *<br>**Drug:** ART x 90 days	Clinician **	ART clinic
	Months 3 and 9	Adherence support<br>Symptom screening for TB, STI<br>Provision of Health education & provision of condoms<br>**Drugs**: Dispensation of 3-monthly pre-packed drugs<br><br>Patients with symptoms referred to clinic	CHiP (community HIV care provider)	Participants Home
	Months 6 and 12	**Clinical visit:** clinical consultation, adherence support[!],<br>**Laboratory:** VL sample collection[ⱡⱡ]<br>**Drugs:** ART refill x 90 days<br><br>VL > 1000 or symptom screening positive – participant down referred to SoC [¥]	Clinician **	ART clinic
Adherence clubs (AC)	Month 0 (enrolment)	Clinical consultation, screening for eligibility*, consent to participate in study *<br>**Drug:** ART x 90 days<br>Club venue and meeting date provided	Clinician**	ART clinic
	Months 3 and 9	**Club meeting consisting of 15-30 participants**<br>Adherence support<br>Symptom screening for TB, STI<br>Provision of Health education & provision of condoms<br>**Drugs**: Dispensation of 3-monthly pre-packed drugs<br><br>Patients with symptoms referred to clinic	CHiP (community HIV care provider)	Community venue (church halls, school classrooms)
	Months 6 and 12	**Clinical visit:** clinical consultation, adherence support[!],<br>**Laboratory:** VL sample collection[ⱡⱡ]<br>**Drugs:** ART refill x 90 days<br><br>VL > 1000 or symptom screening positive – participant down referred to SoC [¥]	Clinician**	ART clinic

*Screening for eligibility and consenting was done by research nurse in the health care facility
**clinician includes physicians, clinical officers and nurses
*** ART refills in the SoC varied from 1-3 monthly supplies depending on drug stocks
! adherence support in the SoC was done either by the clinician or adherence support workers
ⱡ VL testing in the SoC would be done at either one of the 4 visits depending on their annual due date
ⱡⱡ VL testing in intervention arms were done either at the 6 month or 12 month clinical visit if that visit was close to or corresponded to their scheduled VL testing date.
¥ patients in the intervention arms with VL > 1000 copies were notified of their results either during their clinical visit or even called prior to their clinical or community visit to be seen by the clinician at the HCF.

Appendix 2 : Sensitivity analysis showing estimated and risk difference of viral suppression over time among all patients.

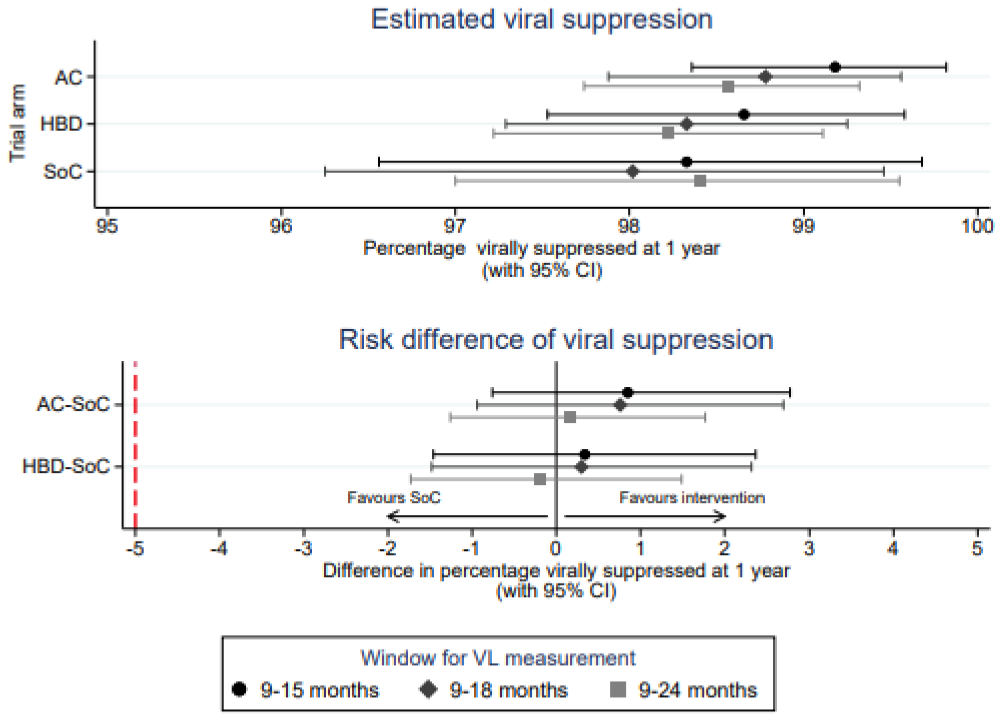

Appendix 3. Composite outcomes (known viral suppression)

	Enrolled and did not transfer out	Known virally suppressed at 12-months*	Estimated prevalence of known viral suppression†	Risk difference vs SoC
Standard of Care	773	384 (49·7%)	50·3% (45·5%, 55·2%)	
Home Based Delivery	835	512 (61·3%)	62·3% (57·6%, 67·1%)	12·0% (5·3%, 18·7%)
Adherence Clubs	844	481 (57·0%)	57·1% (50·9%, 63·2%)	6·7% (-0·9%, 14·4%)

Data are n/N (%).
*Percentages are as per the raw data (the window period 9-15 months after enrolment)
† Means of cluster prevalence by zone

Appendix 4. Risk difference of viral suppression stratified by sex, age group and years on ART.

	Enrolled (N)	No. of those with VL result	VL>1000 copies/ml	Estimated prevalence of viral suppression	Risk difference vs SoC
**Men**					
Standard of Care	226	102 (45·1%)	2	98·2% [95·0%, 100%]	
Home-Based Delivery	247	147 (59·5%)	4	96·5% [92·5%, 99·6%]	-1·72% [-6·30%, 2·86%]
Adherence Clubs	259	148 (57·1%)	3	98·4% [96·4%, 100%]	0·19% [-3·17%, 3·56%]
**Women**					
Standard of Care	555	288 (51·9%)	4	98·4% [96·2%, 100%]	
Home-Based Delivery	605	371 (61·3%)	2	99·5% [98·8%, 100%]	1·15% [-0·59%, 3·47%]
Adherence Clubs	597	337 (56·4%)	1	99·7% [99·1%, 100%]	1·31% [-0·37%, 3·55%]
**Aged under 40**					
Standard of Care	370	182 (49·2%)	4	97·7% [95·2%, 99·7%]	
Home-Based Delivery	434	253 (58·3%)	3	98·9% [97·4%, 100%]	1·21% [-1·28%, 3·95%]
Adherence Clubs	422	226 (53·6%)	0	100% [N/A]*	2·32% [0·34%, 4·76%]
**Aged 40+**					
Standard of Care	411	208 (50·6%)	2	99·2% [97·8%, 100%]	
Home-Based Delivery	418	265 (63·4%)	3	98·9% [97·5%, 100%]	-0·30% [-2·03%, 1·47%]
Adherence Clubs	434	259 (59·7%)	4	98.5% [97·0%, 99·7%]	-0.67% [-2·49%, 1·22%]
**On ART less than 3 years**					
Standard of Care	238	119 (50·0%)	4	94·0% [86·2%, 99·2%]	
Home-Based Delivery	253	164 (64·8%)	3	98·2% [96·0%, 100%]	4·12% [-1·66%, 12·2%]
Adherence Clubs	257	136 (52·9%)	2	98·8% [96·9%, 100%]	4·72% [-0·89%, 12·8%]
**On ART 3+ years**					
Standard of Care	543	271 (49·9%)	2	99·3% [98·2%, 100%]	
Home-Based Delivery	599	354 (59·1%)	3	99·0% [97·8%, 100%]	-0·3% [-1·89%, 1·17%]
Adherence Clubs	599	349 (58·3%)	2	99·2% [97·9%, 100%]	-0·1% [-1·62%, 1·35%]

Data are n (%).*Unable to calculate confidence interval due to no events in AC arm.
